# Credibility of Intentional Reimplantation Techniques for Periodontally Compromised Teeth: A Report of Two Cases

**DOI:** 10.7759/cureus.90821

**Published:** 2025-08-23

**Authors:** Satarupa Suklabaidya, Ilakiya Mathi, Kennedy Babu, Gandhimadhi D, Manoj Margabandhu

**Affiliations:** 1 Department of Periodontology, Mahatma Gandhi Postgraduate Institute of Dental Sciences, Pondicherry, IND

**Keywords:** biomesh, intentional reimplantation, palatoradicular groove, pathological migration, perioglas

## Abstract

Intentional reimplantation is a cautious procedure consisting of the deliberate extraction of an endodontically treated tooth, followed by the examination and manipulation of the root surface and the careful reinsertion of the tooth back into the socket. The main aim of attempting such an advanced and risky procedure is to meet patients' expectations of aesthetics and the retention of their natural dentition. This article tries to shed some light on two different approaches taken to perform this interdisciplinary procedure: to perform intentional reimplantation in a pathologically migrated tooth and in a tooth with a palatoradicular groove (PRG). A 25-year-old male and a 45-year-old female patient reported with mobility along with PRG with respect to tooth number 22 and pathological migration in relation to (irt) tooth number 11, respectively. Endodontic management, followed by intentional reimplantation, was done in both cases, with additional sealing of PRG, guided tissue regeneration (GTR) with bone graft, injectable platelet-rich fibrin (iPRF), and resorbable membrane in the first case. Intentional reimplantation served as a dependable and meticulous option for periodontally compromised cases, as well as for the functional and conservative management of an asymptomatic, malpositioned, pathologically migrated tooth.

## Introduction

The goal of purposeful tooth reimplantation is to salvage a natural tooth that is considered lost after endodontic manipulation [1]. The primary benefit of this method is the accessibility and visibility of all tooth surfaces without endangering nearby periodontal tissues.

Palatoradicular groove (PRG)/palatogingival groove (PGG) is defined as a developmental anomaly starting from the central fossa and extending to a variable distance on the root surface after crossing the cingulum. When present, it is typically located on the maxillary incisor teeth's palatal aspect, the maxillary lateral incisor being most frequently affected owing to potential embryological risk during formation [2,3].

Owing to the anomaly's cervical placement, a palatoradicular groove might offer an avenue for bacterial plaque to build up along the length of the groove and the pulp cavity/auxiliary canals, thus facilitating further contact between the periodontium and pulp canal, resulting in mixed endodontic periodontal diseases.

The first patient in this case report had an anatomically complex lateral incisor with a type II palatoradicular groove, as per Gu's classification [4,5]. A combined treatment strategy was undertaken that includes purposeful replanting, in addition to endodontic therapy. The second case was that of a lady in her late 40s with pathological migration. Intentional reimplantation served to be the most appropriate option after considering her age, as well as her desire to keep her natural tooth.

The rationale of the case report is to shed light on two very distinct approaches taken for intentional reimplantation; since it is typically used as a last resort and is not commonly carried out, there is currently no agreement on the treatment regimen. Numerous investigations have been reported utilizing a variety of methods to protect the natural tooth. It is thought that carrying out every stage with the highest level of accuracy ensures that the treatment is successful.

## Case presentation

Case 1

**Figure 1:**
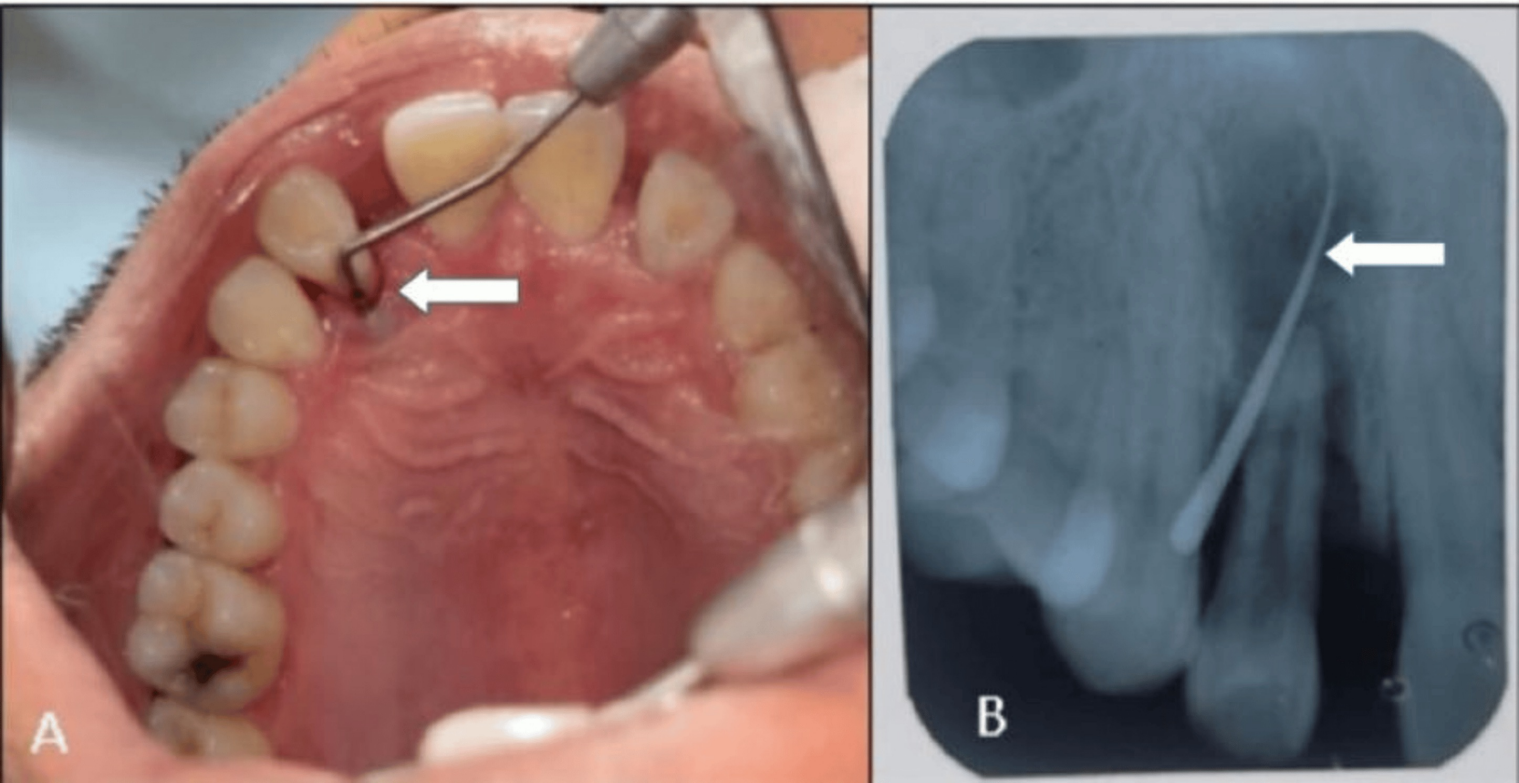
(A) Preclinical examination: the arrow showing a deep periodontal pocket in the palatal aspect of tooth number 22. (B) Intraoral periapical radiograph (IOPAR): the arrow showing gutta percha point in the periapical area irt tooth number 22, which confirms the presence of a sinus tract irt: in relation to

A 25-year-old male patient with no relevant medical history presented with a loose upper maxillary lateral incisor for two years. After a detailed clinical examination, grade 2 mobility, with circumferential periodontal pocket depth of 9 mm, was found; a sinus tract was present labially, along with a palatogingival groove present palatally with respect to the upper left lateral incisor, tooth number 22 (Figure 1A, 1B).

Table 1 provides a comprehensive summary of the chronology of steps undertaken for treating Case 1.

**Table 1:** A comprehensive summary of Case 1 irt, in relation to; PRG, palatoradicular groove; GIC, glass ionomer cement; IOPAR, intraoral periapical radiograph

Case number	Tooth involved	Initial diagnosis	Treatment steps	Follow-up
Case 1	Upper maxillary lateral incisor, tooth number 22	Palatoradicular groove irt 22 with mixed endodontic periodontal infection	1. Root canal treatment	After one week, the sinus tract was closed
			2. Atraumatic extraction of tooth number 22	After two weeks, soft tissue healing was satisfactory
			3. Scaling and root planing irt 22; the tooth should be immersed in 0.9% saline during the extraoral manipulation	After one month, splinting was in place; mobility irt tooth number 22 was substantially reduced
			4. Sealing of PRG with type II GIC	After six months, IOPAR irt 22 shows radiographic bone fill. The splint was still in place. No abnormalities were seen in soft tissue
			5. Reimplantation of tooth number 22 back into the socket with bone graft and non-resorbable membrane	
			6. Splinting	

Treatment Plan

The patient was informed about the poor prognosis of the tooth. But the patient insisted on preserving his natural tooth. Cone beam computed tomography (CBCT) revealed a palatoradicular groove extending beyond the middle third of the root.

As the treatment of PRG is about sealing the groove completely, to get proper access and visibility of the extent of the groove, intentional reimplantation was decided as the treatment of choice.

Surgical Treatment

Treatment was done in two phases: in the first appointment, thorough oral prophylaxis was done, followed by root planing in relation to (irt) tooth number 22 after one week. Proper oral hygiene instructions were given, along with a 0.12% mouth rinse, to use twice a day for two weeks. After two weeks, the patient was followed up; the pocket depth still persisted.

**Figure 2:**
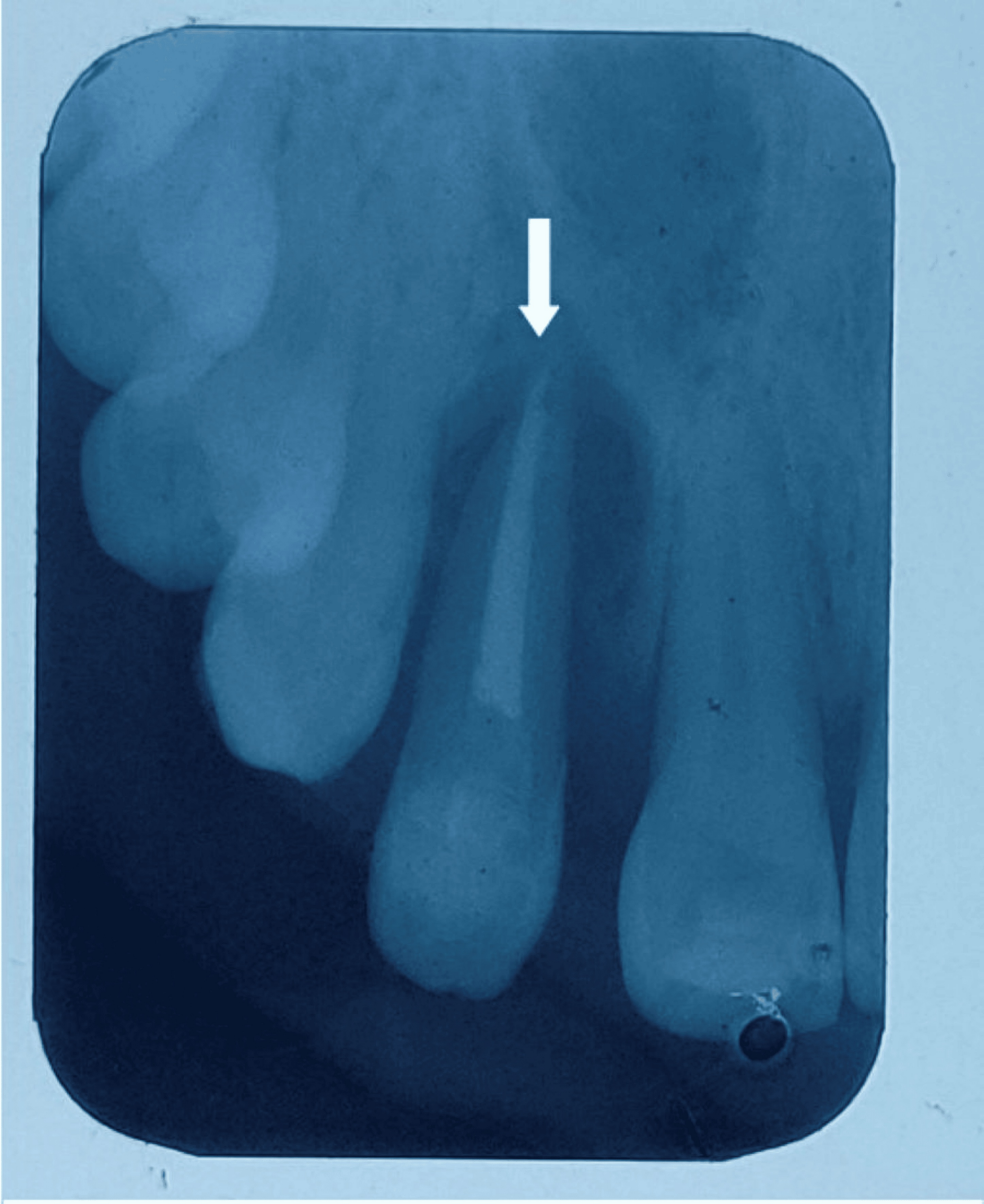
Radiograph after one month of root canal therapy: the arrow showing less amount of radiolucency in the periapical aspect of tooth number 22, indicating that healing has taken place after endodontic therapy

In the second appointment, endodontic access was obtained irt tooth number 22 under local anesthesia (LA). Cleaning and shaping of the canal were carried out using a nickel-titanium (NiTi) ProTaper (Dentsply Sirona, Charlotte, NC). Obturation was done in the lateral condensation technique, following post-endodontic restoration (Figure 2).

**Figure 3:**
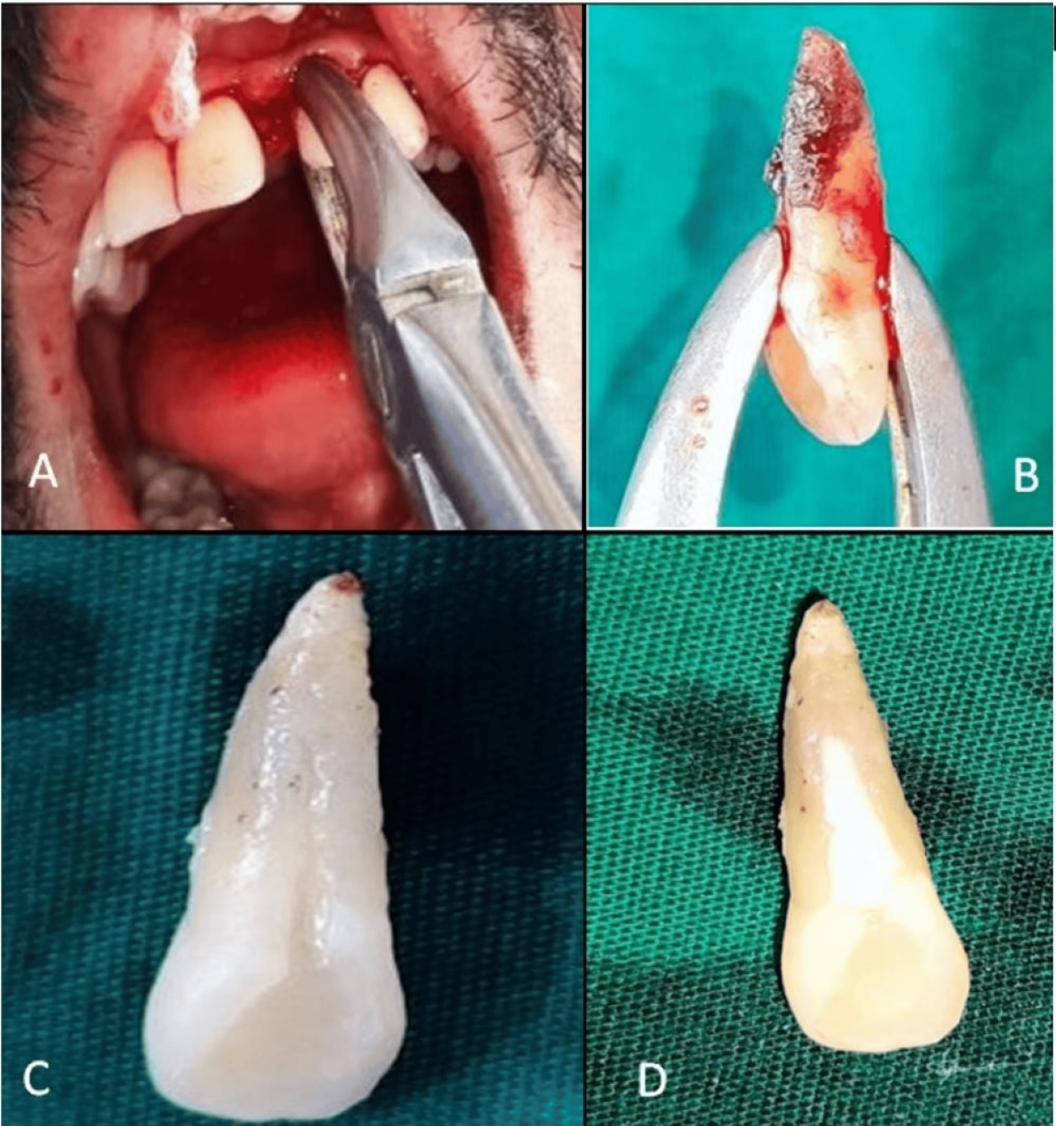
(A) Atraumatic extraction of 22; (B) 22 after extraction; (C) after oral prophylaxis, PRG visible; and (D) sealing of PRG with type II GIC PRG, palatoradicular groove; GIC, glass ionomer cement

After one month, the patient was followed up; the sinus tract and pocket depth still persisted. The surgical phase was started. Under LA, a full-thickness mucoperiosteal flap was elevated on both the palatal and buccal sides. Anterior forceps was applied to tooth number 22, and atraumatic extraction was performed with rotatory movements, avoiding buccolingual movements. Scaling and root planing was done along with copious irrigation using a 0.9% NaCl solution, after which the tooth was placed in a kidney tray containing 0.9% NaCl solution until reimplantation was done. The palatoradicular groove was seen to extend until the apical third, with no associated cracks or abnormalities. Thorough scaling was done to expose the groove. The tooth was held gently by crown and root with physiological saline-soaked gauze during the extraoral procedure. The groove was seen to be type II, so it was decided to seal the groove with type II glass ionomer cement (GIC) (Figure 3).

**Figure 4:**
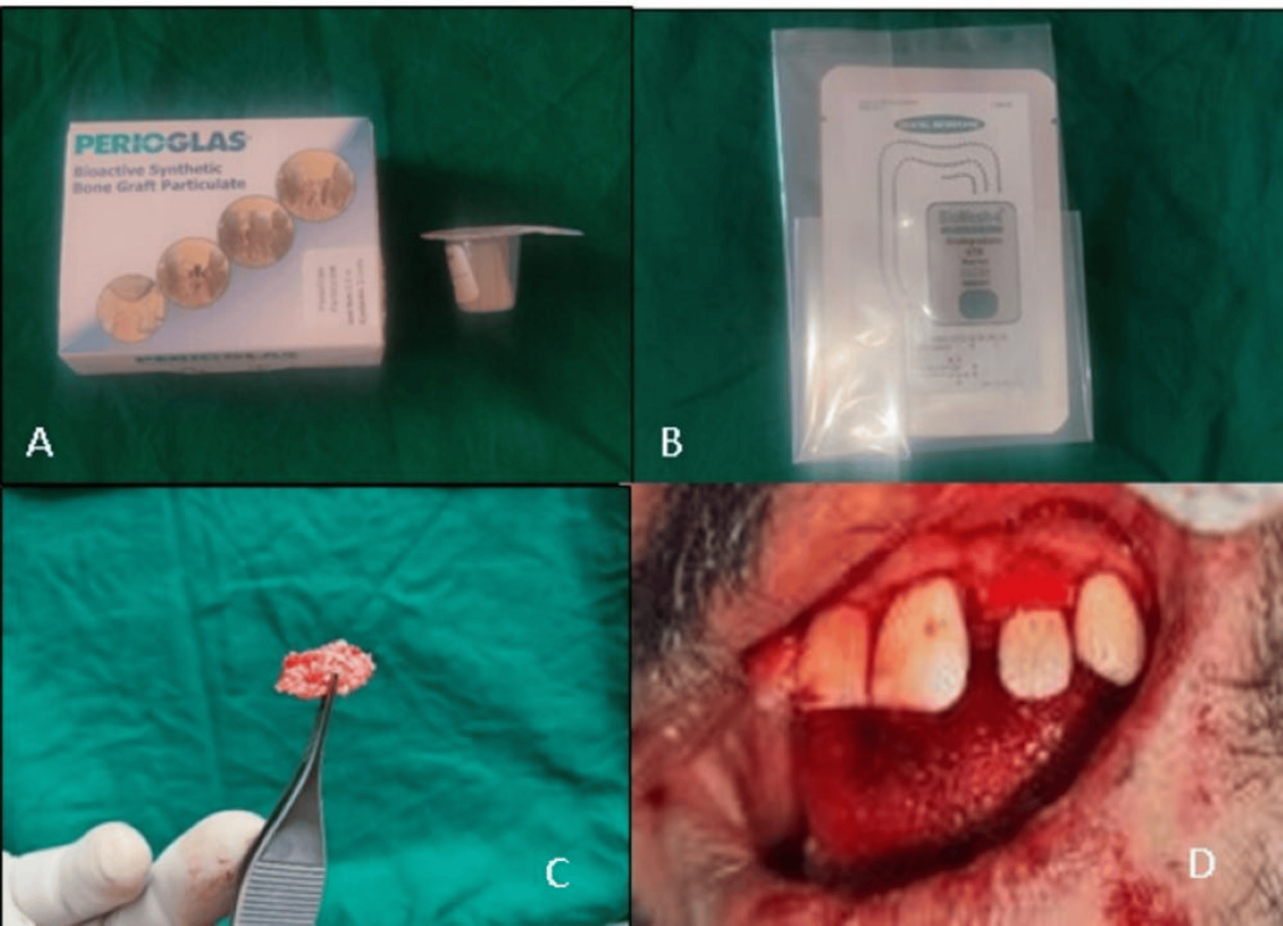
(A) PerioGlas, (B) BioMesh membrane, (C) sticky bone, and (D) PerioGlas placed with BioMesh

The whole extraoral procedure was completed in 10 minutes. The tooth was reimplanted back into the socket, along with bioactive glass (PerioGlas) (0.5 cc) (NovaBone Products, Bangalore, India), and secured with biodegradable BioMesh membrane (Samyang Corporation, Seoul, Korea) (Figure 4).

**Figure 5:**
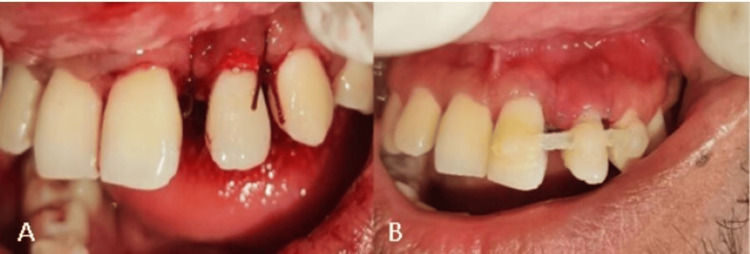
(A) Sutured with 3-0 BBS. (B) Splinting done with a composite splint BBS: black braided silk

The flap was approximated with 3-0 black braided silk (BBS), and splinting was done with a semirigid splint for seven days (Figure 5).

**Figure 6:**
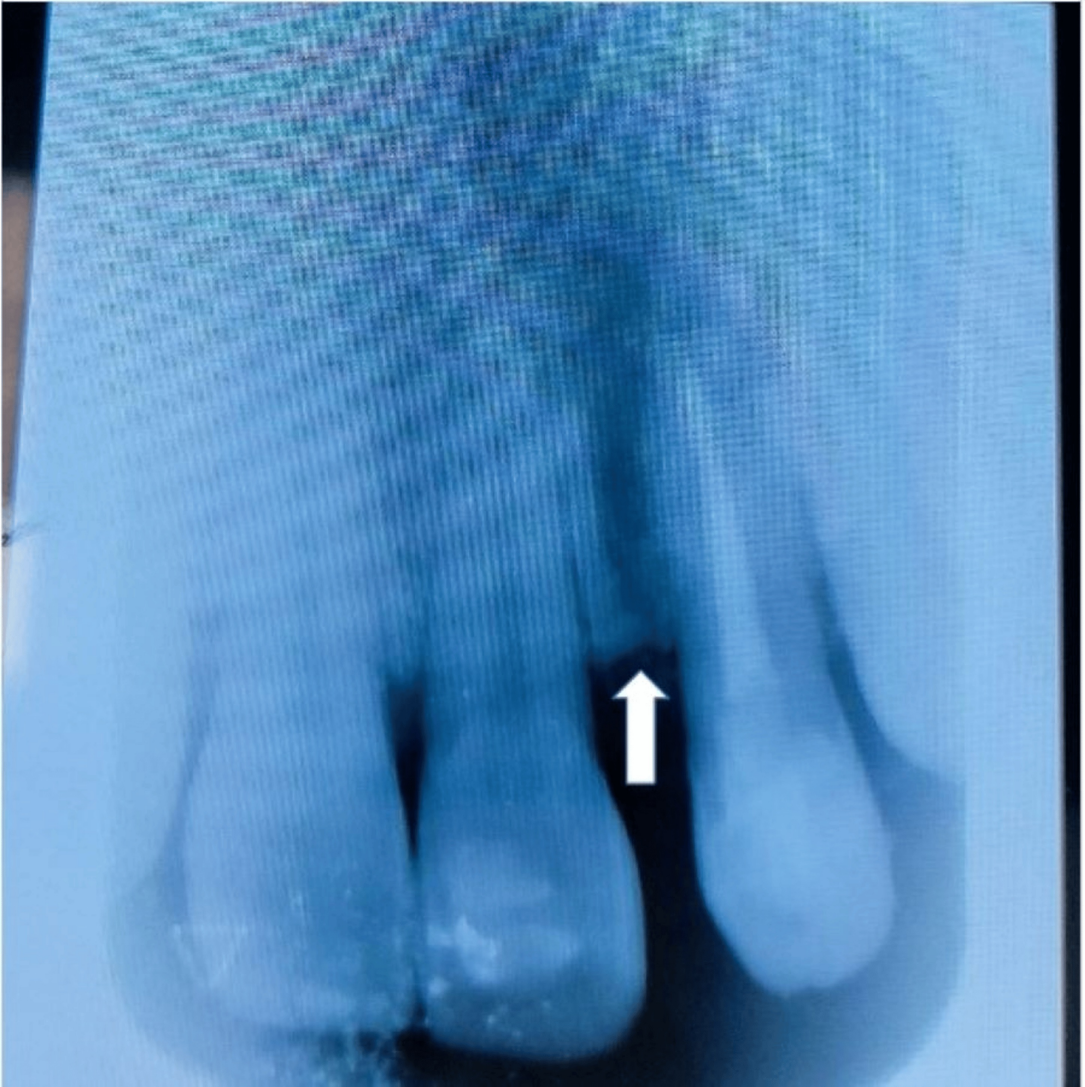
Immediate postoperative radiograph: the arrow showing the bone graft being placed until the alveolar crest

At three months of recall, mobility was reduced to grade 1, and the sinus tract had closed. Pocket depth had reduced to 5 mm. Splinting was decided to continue for another three months (Figure 6).

**Figure 7:**
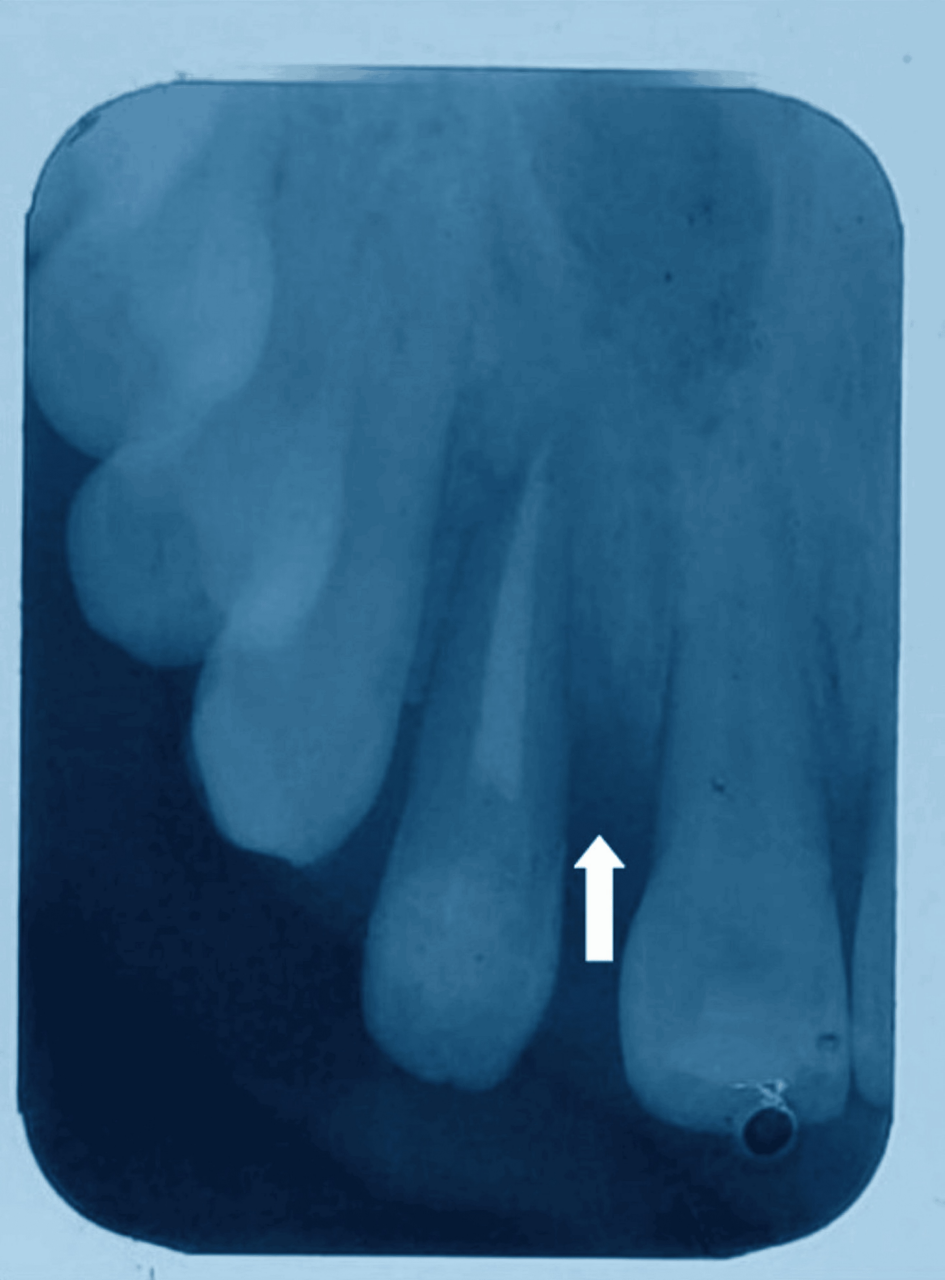
Six-month postoperative radiograph: the arrow showing bone fill being achieved in the mesial aspect of tooth number 22

At six months of recall, the tooth showed complete periapical healing and bone regeneration. The tooth is asymptomatic, and the patient is comfortable (Figure 7).

Table 1 provides a comprehensive summary along with a chronology of steps undertaken for treating the above case.

Case 2

A female patient, aged 45 years, came to the department of periodontics with the chief complaint of a migrated tooth in the front tooth region.

**Figure 8:**
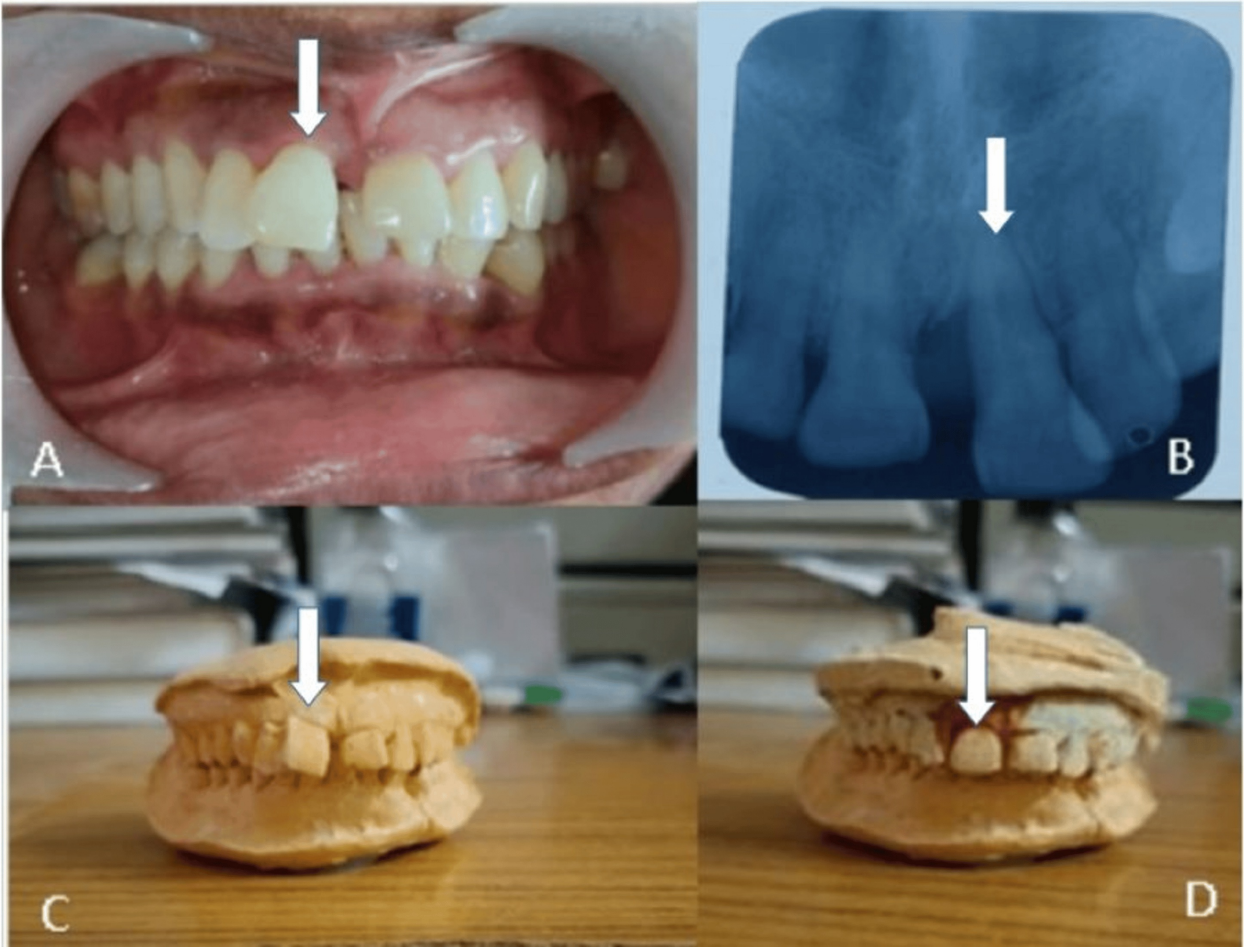
(A) Preclinical examination: the arrow showing pathologically migrated tooth number 11. (B) Preoperative radiograph irt 11: the arrow revealing bone loss present in the mesial aspect but no periapical abnormalities. (C) Study model: the arrow showing tooth number 11 in pathologically migrated position. (D) Study model: the arrow depicting the achievable position of tooth number 11 after intentional reimplantation so as to give the patient a better understanding of the procedure

On examination, tooth number 11 was found to be pathologically migrated with no mobility and a normal probing depth of 4 mm. IOPAR irt tooth number 11 revealed no periapical involvement. The patient wanted aesthetic correction of 11. The patient was informed about the limitations of orthodontic intervention owing to the age of the patient using models (Figure 8).

**Figure 9:**
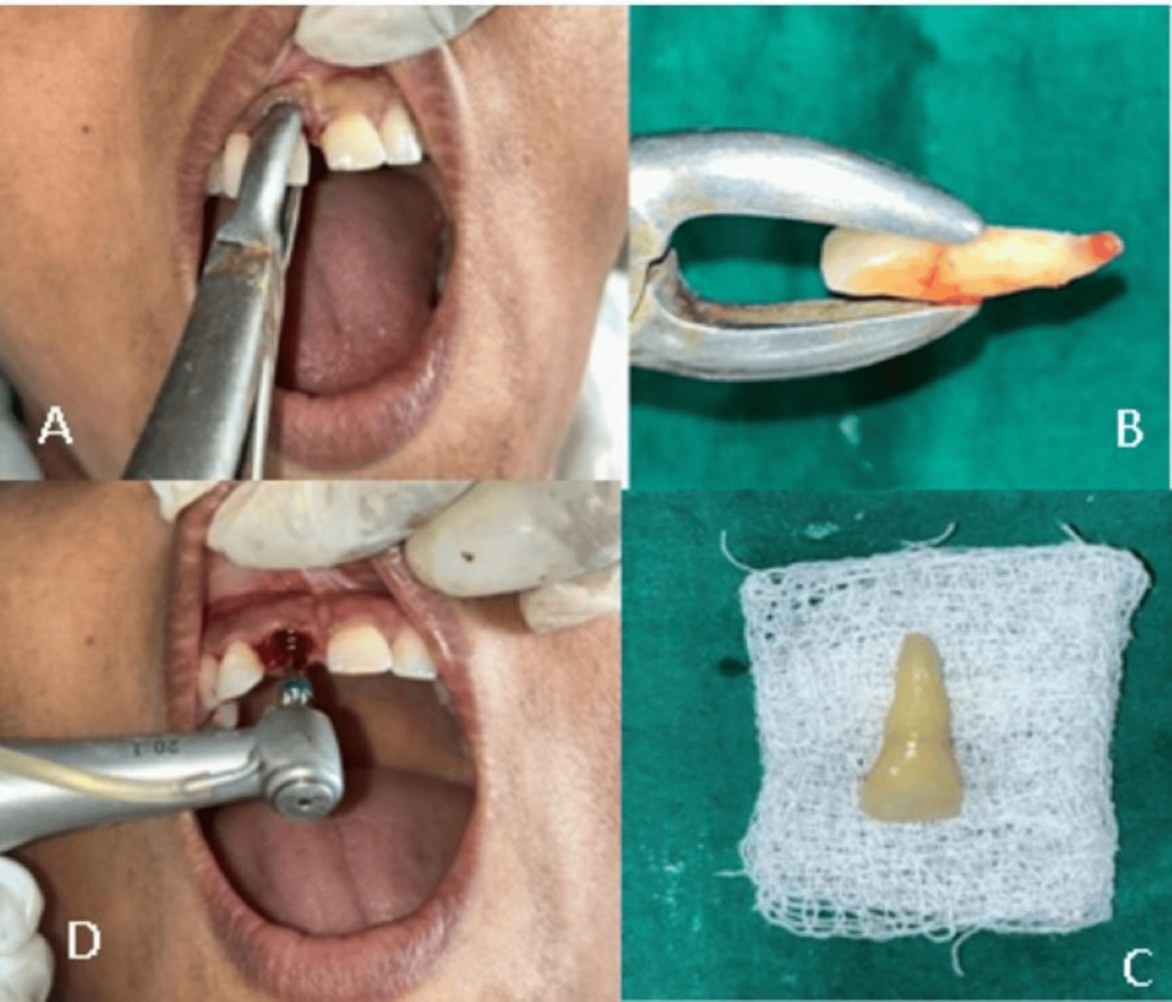
(A) Atraumatic extraction, (B) extracted tooth, (C) after apicoectomy, and (D) corticotomy done

After one month, the surgical phase was started. Antibiotic prophylaxis of amoxicillin 1 g twice per day (bid) for five days was prescribed, along with 0.12% chlorhexidine mouth rinse. Full-thickness mucoperiosteal flap was raised. Atraumatic extraction of 21 was done without using any elevators (Figure 9A, 9B). The tooth was placed immediately in a 0.9% NaCl solution. Thorough scaling and root planing was done for 21. The tooth root end was resected, and a class 1 cavity was made. The root end was restored with GIC. All the extraoral procedure was done while holding the tooth with wet gauze and finished in 10 minutes (Figure 9C). Using drills from the implant kit, corticotomy was done in the apical portion of the socket so as to reorient the tooth in the desired position (Figure 9D).

**Figure 10:**
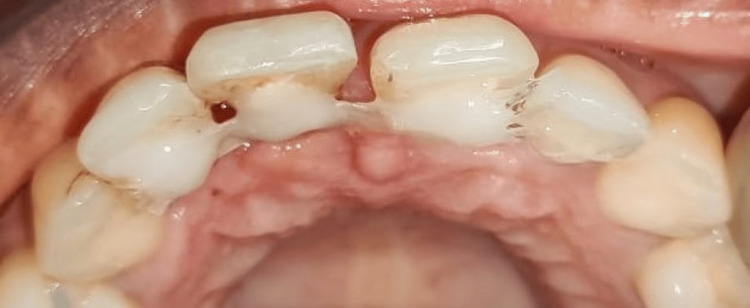
Splinting done

The maxillary bone is usually D3, so <2500 revolutions per minute (rpm) and less than 30 nm torque were used along with copious irrigation with chilled saline. The teeth were reimplanted into a new position and stabilized with a semirigid splint for seven days (Figure 10).

**Figure 11:**
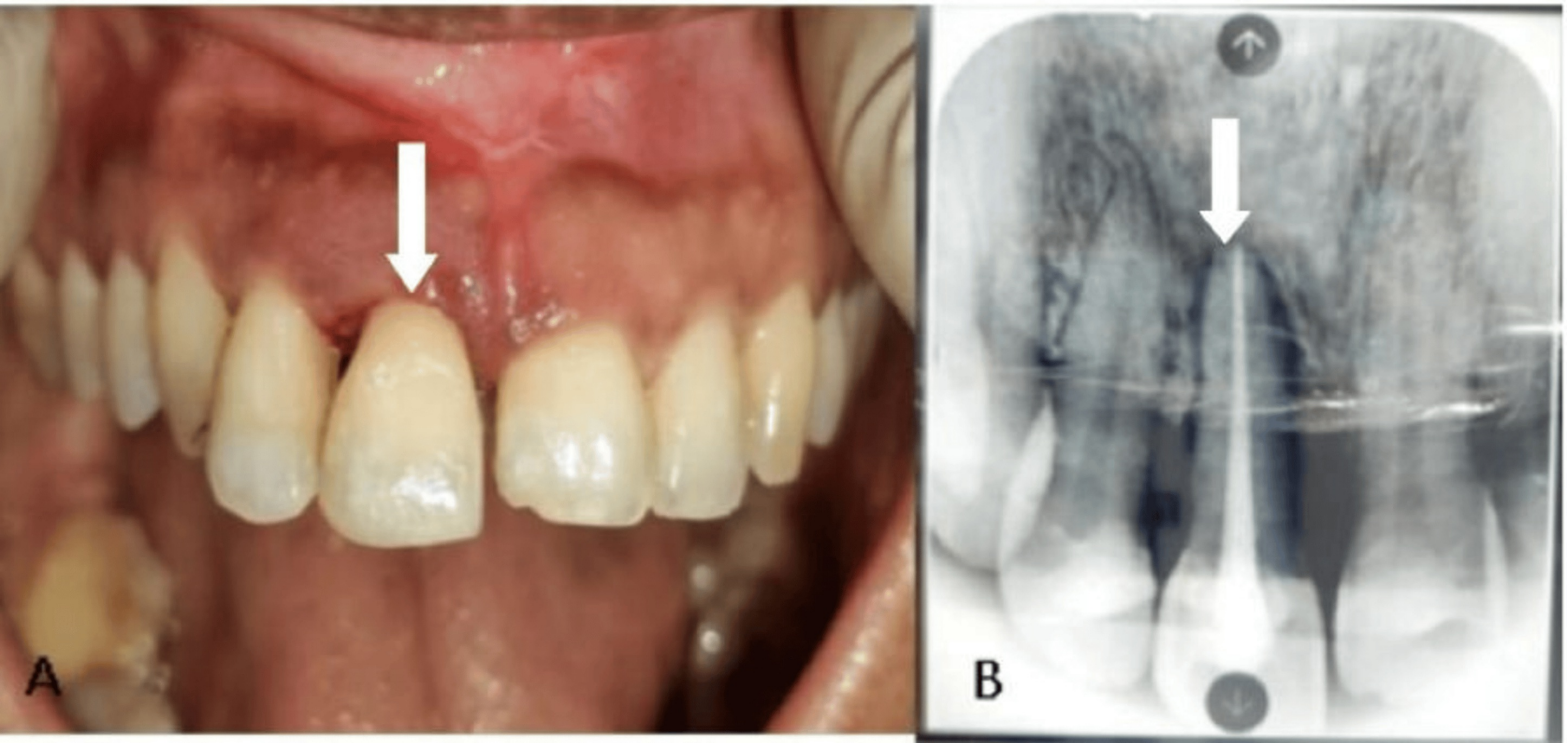
(A) Six-month postoperative clinical photograph: the arrow showing reimplanted tooth number 11 stable in the present position. (B) Six-month postoperative radiograph: the arrow showing bone formation around the reimplanted tooth with no periapical abnormalities

On a six-month evaluation, both clinical and radiographic photos showed satisfactory results (Figure 11).

Table 2 provides a comprehensive summary along with a chronology of steps undertaken for treating Case 2.

**Table 2:** A comprehensive summary of Case 2 irt, in relation to; IOPAR, intraoral periapical radiograph

Case number	Tooth involved	Initial diagnosis	Treatment steps	Follow-up
Case 2	Upper maxillary right central incisor, irt 11	Pathologic migration irt 11	1. Root canal treatment	After one week, soft tissue healing was satisfactory
			2. Atraumatic extraction of tooth number 11	After one month, reimplanted tooth was stable. Splinting was in position. No soft tissue abnormality was seen
			3. Scaling and root planing irt 22; the tooth should be immersed in 0.9% saline during the extraoral manipulation	After six months, IOPAR reveals bone formation around the apex. No signs of mobility or inflammation seen
			4. Corticotomy done irt 11	
			5. Splinting done	
			6. Composite restoration done irt 21	

## Discussion

Procedures such as intentional reimplantation may be the sole option in situations such as this, when traditional treatment is not a practical alternative.

According to a literature analysis by Torabinejad et al., the success rate for purposefully replanted teeth is as high as 95%, with an overall survival rate of 88% [6].

In order to maintain the viability of the periodontal ligament (PDL) cells and start the regeneration process, the extraoral time following tooth extraction before reinsertion is crucial. It is important to use a storage medium to maintain and improve the viability of PDL fibroblasts in an avulsed tooth when a quick replantation is not possible at that particular time; 0.9% NaCl has been used in both cases as it is found to be compatible with cells of PDL, though lacking essential nutrients. Numerous experts have emphasized how crucial it is to protect PDL and keep extraoral time to a minimum of 15-20 minutes [7].

Root resorption and ankylosis are the two outcomes that can be expected as results of intentional reimplantation. Long extra-alveolar periods and the loss of pericementum influence replacement resorption, whereas infection following an incorrect root canal treatment (RCT) results in inflammatory resorption [8]. Fortunately, we have not encountered any such scenario in both cases until now.

The regenerative process is facilitated by the requirement for atraumatic extraction that avoids fracturing the socket walls. This can be accomplished by limiting the extraction force applied to the tooth's crown [9].

The root surfaces should be handled with minimal manipulation [10]. Root planing demonstrated the cementum's resistance to the resorptive process, which would otherwise be triggered by the remaining necrotic PDL fragments. Therefore, after tooth reimplantation, the removal of necrotic PDL material from the root surface greatly slowed down tooth resorption [11].

Corticotomy was found to be helpful in an effort to promote the osteoinduction process [12].

The selection of GIC for sealing the palatoradicular groove was based on its capacity to withstand water degradation at the tooth-cement interface, its strong chemical bonding ability, and its antibacterial properties. The fluoride in GIC may prevent the adhesion of bacteria first and may also impede their growth and metabolism. Additionally, it has been noted that the cement surface was the site of epithelial and connective tissue attachment [13].

After a nine-month follow-up, Rajendran and Sivasankar observed that platelet-rich fibrin (PRF) application combined with bone grafting on the bony defect area led to the production of crestal bone in PGG patients [14]. Gandhi et al. also documented the use of synthetic bone graft material in a large PRG-related bone defect area [15]. Bioactive glass was selected for this case because of its ease of handling and manipulation, considerable reduction in junctional epithelium migration at the treated intrabony defect (IBD) sites, and capacity to attach to both soft tissues and the bone [16].

PerioGlas is made of calcium silicate bioactive glass, which combines with surrounding tissues to initiate osteostimulatory effect and functions as an osteoconductive bone transplant bioactive material. When compared to autograft, bioactive glass is regarded as the second-best graft material.

Approximately 62% of cases treated with resorbable barriers showed therapeutic success during a six-month follow-up, according to Anderegg and Metzler's findings [17]. BioMesh is a resorbable membrane made of polylactic acid (PLA), polyglycolic acid (PGA), and lactide/glycolide copolymer (poly(lactide-co-glycolide) {PLGA}).

Injectable PRF (iPRF) was used in our case to form sticky bone by combining PRF with PerioGlas to form a durable fibrin bone graft that is manageable and moldable into the required shape. The "low speed concept" for blood centrifugation was introduced by Ghanaati et al. with the intention of creating iPRF, a liquid formulation of PRF devoid of fibrin matrix or anticoagulants. It was shown that a greater number of cells, including leukocytes, were present at lower centrifugation rates before a fibrin clot formed [18]. Chang et al. state that PRF enhances osteoprotegerin (OPG) and phosphorylated extracellular signal-regulated protein kinase (P-ERK) expression, both of which lead to osteoblast proliferation and possible bone production [19].

From the introduction of this procedure, there has unavoidably been discussion on the impact of splinting kind and length on the healing of a replanted tooth. Despite the fact that it has been predicted accurately that prolonged splinting may result in root resorption or ankylosis, nevertheless, there has been no evidence to support a shorter healing period. A case report by Demiralp et al. found that after splinting the reimplanted tooth for up to three months, there were no radiographic indications of ankylosis or root resorption [20].

In the first case, we maintained the intraoral splint for six months due to an existing deteriorating periodontium, a large bone defect, and moderate improvement in tooth mobility. On the other hand, the second case's reimplanted central incisor was comparatively firm by three months, and the tooth that was previously movable in grade 2 now moved within physiological bounds by six months. Regarding the radiographic bone fill, the first case demonstrated moderate bone fill.

Both cases showed no postoperative signs of excessive mobility, root resorption, or an abnormal clinical crown-root ratio that would raise aesthetic problems. However, the first case did show some amount of residual mobility after six months of review, because of a large amount of periapical and periodontal destruction that was present preoperatively. It is expected to be completely stable within the next six months. No ankylosis was seen in both cases. Additionally, both patients were happy because their needs for maintaining their natural teeth, time and money limits to choose other restorative procedures that are unquestionably effective, and aesthetics were all met.

Table 3 gives a comparative assessment of intra- and postoperative challenges faced in both cases.

**Table 3:** A comparative assessment of intraoperative and postoperative challenges of Cases 1 and 2

Case number	Intraoperative challenges	Postoperative challenges
Case 1	Extraoral dry time (should be less than 15 minutes)	Splinting (splinting had to be done labially, making the process a bit difficult and time-consuming)
Case 2	Corticotomy: the position and angulation of the drill used have to be precise to avoid any perforation of the palate	Aesthetic concern: after reimplanting the tooth in the desired position, a composite restoration had to be done for the adjacent tooth
	Extraoral dry time	

## Conclusions

When traditional therapy is ineffective, intentional reimplantation offers a glimmer of hope. It assists in addressing patient issues that are occasionally disregarded during standard procedures. With the careful management of the surrounding tooth structures, it can also be tried in cases where the periodontal tissue is compromised. It is also possible to mix several regenerative techniques to achieve beneficial results. One such attempt to handle teeth with a terminal prognosis with satisfactory patient acceptability is illustrated by this report of two patients.
